# Rechargeable Solid‐State Na‐Metal Battery Operating at −20 °C

**DOI:** 10.1002/advs.202302774

**Published:** 2023-07-23

**Authors:** Haibo Jin, Xiong Xiao, Lai Chen, Qing Ni, Chen Sun, Runqing Miao, Jingbo Li, Yuefeng Su, Chengzhi Wang

**Affiliations:** ^1^ Beijing Institute of Technology School of Materials Science and Engineering Beijing Key Laboratory of Construction Tailorable Advanced Functional Materials and Green Applications Beijing Key Laboratory of Environmental Science and Engineering Beijing 100081 China; ^2^ Beijing Institute of Technology Chongqing Innovation Center Chongqing 401120 China

**Keywords:** interfacial resistance, low‐temperature operation, Na‐metal batteries, NASICON‐type solid electrolyte

## Abstract

Achieving satisfactory performance for a solid‐state Na‐metal battery (SSNMB) with an inorganic solid electrolyte (SE), especially under freezing temperatures, poses a challenge for stabilizing a Na‐metal anode. Herein, this challenge is addressed by utilizing a Natrium super ionic conductor (NASICON) NASICON‐type solid electrolyte, enabling the operation of a rechargeable SSNMB over a wide temperature range from −20 to 45 °C. The interfacial resistance at the Na metal/SE interface is only 0.4 Ω cm^2^ at 45 °C and remains below 110 Ω cm^2^ even at −20 °C. Remarkably, long‐term Na‐metal plating/stripping cycles lasting over 2000 h at −20 °C are achieved with minimal polarization voltages at 0.1 mA cm^−2^. Further analysis reveals the formation of a uniform Na_3−_ *_x_* Ca*_x_* PO_4_ interphase layer at the interface, which significantly contributes to the exceptional interfacial performance observed. By employing a Na_3_V_1.5_Al_0.5_(PO_4_)_3_ cathode, the full battery system demonstrates excellent adaptability to low temperatures, exhibiting a capacity of 80 mA h g^−1^ at −20 °C over 50 cycles and retaining a capacity of 108 mAh g^−1^ (88.5% of the capacity at 45 °C) at 0 °C over 275 cycles. This research significantly reduces the temperature threshold for SSNMB operation and paves the way toward solid‐state batteries suitable for all‐season applications.

## Graphical abstract

By tuning the intrinsic feature of a Natrium super ionic conductor (NASICON) NASICON‐type solid electrolyte via calcium ion (Ca^2+^) doping to fulfill record‐low interfacial resistances of 0.4 Ω cm^2^ at 45 °C and 110 Ω cm^2^ at −20 °C, a rechargeable solid‐state Na‐metal battery operating in a wide temperature range from −20 to 45 °C to promote the concept of all‐season applicable solid‐state batteries is achieved.

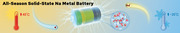

## 1 Introduction

Rechargeable sodium‐based batteries have garnered significant attention as a promising technology for cost‐effective energy storage and power devices in the battery market.^[^ ^1^, ^2^ ^]^ Among them, solid‐state sodium (Na)‐metal batteries (SSNMBs) utilizing a solid electrolyte (SE) have emerged as a viable solution to address safety concerns associated with sodium‐ion batteries (NIBs) employing a liquid electrolyte (LE).^[^ ^3^, ^4^, ^5^ ^]^ The development of SE materials that efficiently conduct Na^+^ ions, encompassing ceramics, polymers, and their hybrid composites, has been crucial for the advancement of SSNMBs. Presently, sodium‐ion SEs based on oxides,^[^ ^6^, ^7^, ^8^ ^]^ sulfides,^[^ ^9^, ^10^, ^11^ ^]^ and polymer composites^[^ ^12^, ^13^, ^14^ ^]^ exhibit high conductivities ranging from 10⁻⁴ to 10⁻^3^ S cm⁻¹ at 25 °C, approaching those of traditional ester‐ or ether‐based liquid electrolytes. However, many SSNMBs still encounter challenges such as low Coulombic efficiency, inferior rate performance, and limited cycling life, primarily due to substantial resistance and poor compatibility at the electrode/SE interfaces.^[^ ^15^, ^16^, ^17^ ^]^ Furthermore, operating SSNMBs within a seasonally varying temperature range from −20 to 40 °C remains a significant hurdle, attributed to sluggish charge‐transfer kinetics at the solid–solid contact interfaces within SSNMBs under low temperatures.^[^ ^18^, ^19^ ^]^ Considering practical application scenarios, there is a pressing need to explore effective strategies that enhance interface kinetics and reduce the operating temperature threshold of SSNMBs.^[^ ^20^, ^21^ ^]^

In recent years, significant efforts have been directed toward SSNMBs utilizing a Natrium super ionic conductor (NASICON) NASICON‐type Na₃Zr₂Si₂PO₁₂ (NZSP) SE.^[^ ^22^ ^]^ Various interface–interphase engineering methods have been proposed to address the challenges of Na⁺‐ion transport, suppression of Na‐metal dendrite formation, and long‐term cycling stability of SSNMBs under ambient temperatures.^[^ ^22^ ^]^ Among these methods, chemical surface modification has proven effective in improving the interfacial performance at the Na‐metal/SE interface.^[^ ^23^, ^24^, ^25^, ^26^ ^]^ For instance, Miao et al.^[^ ^23^ ^]^ achieved homogeneous Na‐metal plating/stripping cycles lasting 5000 h at 25 °C under a high current density of 0.3 mA cm⁻^2^ by introducing a CuO‐coating layer as an active interphase to accommodate the Na/NZSP interface. Conversely, precise control over in situ interphase formation has demonstrated unique advantages in tuning interfacial chemistry to overcome limitations. Li et al.^[^ ^27^ ^]^ manipulated the interface chemistry of a Na₃.₄Zr₁.₈Cu₀.₂Si₂PO₁₂ SE by incorporating Cu^2^⁺/Cu⁺ redox, resulting in a favorable interphase layer of Cu₃PO₄, thereby facilitating a NaCrO₂‐based SSNMB with long‐term cycling performance at 25 °C. Ni et al.^[^ ^20^ ^]^ employed electrochemical migration of K⁺ from the cathode side to the anode side to generate an in situ Na–K alloy interphase, thereby promoting kinetics at the Na/NZSP interface and enabling the operation of a K₂MnFe(CN)₆||Na SSNMB at 0 °C. Additionally, methods such as constructing monolithic structures,^[^ ^28^ ^]^ designing grain boundaries,^[^ ^29^, ^30^, ^31^ ^]^ doping the SEs with metal ions,^[^ ^32^ ^]^ and modifying the Na‐metal anode^[^ ^33^ ^]^ have been reported to reduce interfacial resistance and facilitate dendrite‐free Na‐metal plating/stripping behavior. Despite significant progress, SSNMBs are still far from meeting the requirements for practical applications. A comprehensive examination and investigation of the temperature‐dependent electrochemical behavior of SSNMBs using inorganic SEs has been rarely conducted over a wide temperature range encompassing all‐season conditions, particularly temperatures below 0 °C.^[^ ^20^, ^32^ ^]^

In this study, we present the first demonstration of a rechargeable SSNMB operating over a wide temperature range from −20 to 45 °C. We achieved this by incorporating a calcium ion (Ca^2+^)‐doped Na₃Zr₂Si₂PO₁₂ (Ca‐NZSP) SE, which effectively stabilizes the Na‐metal anode and enables smooth Na‐metal plating/stripping cycles even at low temperatures. Through temperature‐resolution electrochemical impedance spectroscopy (TR‐EIS) measurements on a symmetrical Na/Ca‐NZSP/Na cell, we observed an interfacial resistance of only 0.4 Ω cm^2^ at 45 °C, which increased to less than 110 Ω cm^2^ even at −20 °C at the Na metal/SE interface. Galvanostatic charge/discharge cycling experiments on the symmetrical cell further demonstrated stable Na‐metal plating/stripping cycles with minimal polarization voltages at 0.1 mA cm⁻^2^ across temperatures ranging from −20 to 45 °C. Additionally, we successfully maintained steady Na‐metal plating/stripping cycles for over 2000 h at freezing temperatures as low as −20 °C, with no noticeable voltage fluctuations. By assembling an SSNMB using a composite cathode based on Na₃V₁.₅Al₀.₅(PO₄)₃, we achieved excellent cycling performance within the temperature range from −20 to 45 °C. Notably, the battery delivered a reversible capacity of 108 mAh g⁻¹ at 0 °C with over 95% capacity retention after the 275th charge/discharge cycle. This work represents a significant breakthrough in reducing the operating temperature threshold of SSNMBs to encompass extremely cold temperatures and brings us closer to the practical application of solid‐state batteries.

## 2 Results and Discussion

**Figure 1:**
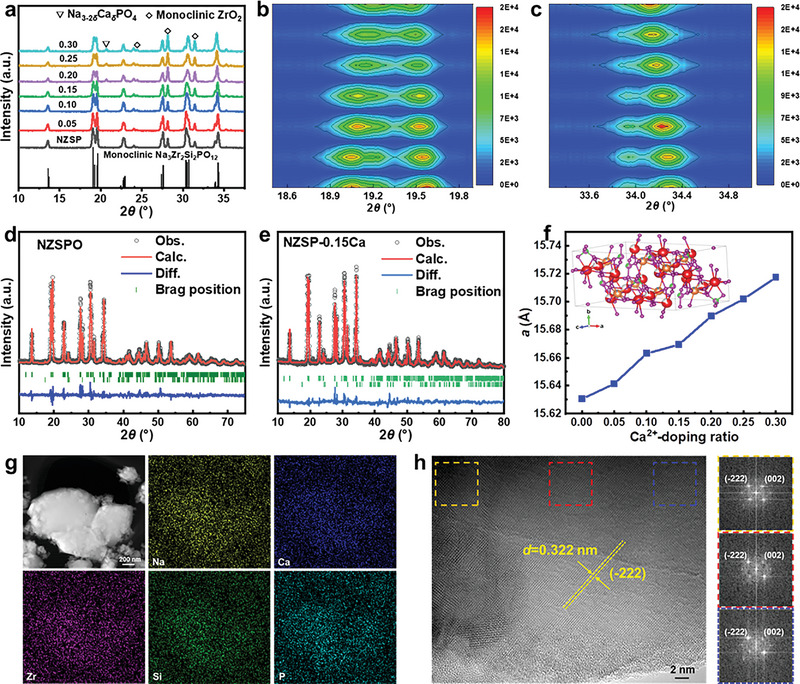
a) XRD patterns of the Na_3+2_ *_x_* Zr_2−_ *_x_* Ca*_x_* Si_2_PO_12_ samples denoted as *x*Ca‐NZSP (*x* = 0–0.3). Contour map diagrams of small‐angle XRD patterns in the selected 2*θ* range of b) 18.5°–20.0° and c) 30.3°–35.0°. Rietveld refinement XRD results of d) NZSP and e) 0.15Ca‐NZSP. f) Relationship of the refined lattice parameter *a* (Å) against the Ca^2+^‐ion doping ratio. Inset the crystal structure of 0.15Ca‐NZSP. g) Dark‐field TEM image of 0.15Ca‐NZSP with corresponding EDS mapping images. h) High‐resolution TEM image with three Fourier‐transform diagrams.

Considering a doping strategy,^[^ ^34^ ^]^ the Ca^2+^ with the promise of regulating the lattice structure and the chemical component of the NZSP SE for an improved conductivity and interfacial features is selected to partially replace the Zr^4+^ for investigation. Samples of Ca‐NZSP with the chemical formulation Na_3+2_ *_x_* Zr_2−_ *_x_* Ca*_x_* Si_2_PO_12_ (noted as *x*Ca‐NZSP, where *x* ranges from 0 to 0.30) were synthesized utilizing the solid‐state reaction technique. We employed raw materials, including Na_2_CO_3_, ZrO(NO_3_)_2_, CaC_2_O_4_, SiO_2_, and NH_4_H_2_PO_4_, and subjected them to sintering in a pure oxygen atmosphere to mitigate the adverse influences of extraneous gases such as carbon dioxide and water vapor. The crystal and phase structures were evaluated employing X‐ray diffraction (XRD), with a copper Kα radiation source. **Figure** **1**a presents the XRD patterns of the *x*Ca‐NZSP samples, each being identified as a multiphase mixture with the predominant monoclinic Na_3_Zr_2_Si_2_PO_12_ phase. Noteworthy diffraction peaks at 2*θ* values of 19.07°, 19.25°, 19.59°, 19.62°, 34.29°, and 34.35° correspond to the (111), (−202), (020), (−311), (−331), and (−602) planes of the monoclinic Na_3_Zr_2_Si_2_PO_12_ phase, respectively. The diffraction peaks recorded at 2*θ* values of 24.40°, 28.15°, and 31.44° can be attributed to a monoclinic ZrO_2_ phase (ICSD#85243), instigated by sodium volatilization under high temperatures.^[^ ^35^, ^36^, ^37^ ^]^ With the increase in the Ca^2+^‐doping ratio exceeding 0.15, an additional diffraction peak emerges at 2*θ* = 20.70°, which can be associated with an extra precipitated phase of Na_3−2_ *_δ_* Ca*_δ_* PO_4_. This implies a solubility threshold for Ca^2+^ ions within the lattice of monoclinic Na_3_Zr_2_Si_2_PO_12_.^[^ ^37^ ^]^ Contour map diagrams of the small‐angle XRD patterns within the selected 2*θ* ranges of 18.5–20.0° and 30.3–35.0° are illustrated in Figure 1b,c, respectively. As the Ca^2+^‐ion doping ratio rises, noticeable shifts in multiple peaks are observed. Specifically, the diffraction peaks corresponding to the (111) and (−202) planes migrate toward higher angles, whereas those of the (020), (−311), (−331), and (−602) planes shift toward lower angles.

The impact of Ca^2+^‐ion doping on the cell structure of Na_3_Zr_2_Si_2_PO_12_ was scrutinized using Rietveld refinement of the XRD patterns. Figure 1d,e delineates the Rietveld refinement outcomes for the undoped NZSP sample and the 0.15Ca‐NZSP, respectively, while results for other samples can be found in Figure S1 (Supporting Information). Figure 1f and Figure S2 (Supporting Information) expound on the correlation between lattice parameters (*a*, *b*, *c*, *β*) and the Ca^2+^‐ion doping ratio. Findings suggest that parameters *a*, *b*, and *β* escalate, whereas *c* diminishes, concurrent with an increase in the Ca^2+^‐ion doping ratio. This cell expansion can be ascribed to the larger ionic radii of Ca^2+^ (0.106 nm) compared to that of Zr^4+^ (0.072 nm) and the augmented concentration of Na^+^‐ions within the lattice for charge compensation. These findings are congruent with earlier literature on Ca‐NZSP.^[^ ^28^, ^38^ ^]^ The morphology and microstructure of the 0.15Ca‐NZSP ceramic particles were analyzed using a scanning electron microscope (SEM) and a transmission electron microscope (TEM) outfitted with an energy‐dispersive spectrometer (EDS). Cross‐sectional SEM images of the samples are included in Figure S3 (Supporting Information). The NZSP displays a low relative density of 73.8%, along with numerous pores and cracks. Ca^2+^‐ion doping augments the densification sintering of the *x*Ca‐NZSP samples, with the highest relative density of 96.2% observed for 0.15Ca‐NZSP. An increase in the Ca^2+^‐ion ratio beyond this point results in a decline in the samples’ density due to the aggregation of significant amounts of low‐density products at the grain boundary when *x* ≥ 0.20.^[^ ^37^ ^]^ Figure 1g presents the dark‐field TEM image of the 0.15Ca‐NZSP particles, alongside corresponding EDS mapping images, suggesting a uniform distribution of Na, Ca, Zr, Si, and P in the 0.15Ca‐NZSP. Figure 1h shows a high‐resolution TEM image and selected Fourier transform diagrams. Clear lattice fringes associated with the (−222) planes and matching diffraction spots are discernible, resonating with a monoclinic NASICON structure. X‐ray photoelectron spectroscopy (XPS) using an Al Kα radiation source was utilized to assess the surface chemical states of the 0.15Ca‐NZSP. The XPS spectra in Figure S4 (Supporting Information) confirm the presence of Na, Zr, Si, P, O, and Ca species, calibrated using the C 1s (C─C binding energy, 284.8 eV). The Ca 2p spectrum, featuring a typical Ca 2p_1/2_ peak at 350.53 eV and a Ca 2p_3/2_ peak at 347.23 eV, verifies the incorporation of doped Ca^2+^ ions. In contrast to the solid electrolytes sintered in the air,^[^ ^26^ ^]^ the 0.15Ca‐NZSP sintered in a pure O_2_ atmosphere displays no evident XPS signals of CO_3_^2−^ in the C 1s spectrum, which contains only a C─C peak at 284.8 eV.

The conductivity of the samples was determined through high‐frequency impedance analysis within the frequency range of 50–10 MHz, utilizing a sputtering Au film as the block electrode. As illustrated in Figure S5 (Supporting Information), the Nyquist plots exhibit uniform attributes, including an intercept on the *Z*_r_ axis at the higher frequency end, followed by a diagonal semicircle and a linear tail. The intercept at the frequency end is posited to denote the bulk grain resistance (*R*_b_); the diameter of the semicircle is believed to correspond to the grain boundary resistance (*R_g_*_b_); and the sum of *R*_b_ and *R_g_*_b_ yields the total resistance (*R*_t_).^[^ ^39^, ^40^ ^]^ Subsequently, the total conductivity (*σ*_t_) can be calculated using the equation: *σ*_t_ = *L*/*R*_t_, where *L* represents the thickness of the disk. The findings reveal an optimal doping ratio of *x* = 0.15, where the 0.15Ca‐NZSP demonstrates a total conductivity of 1.59 mS cm^−1^, a value 4.3 times greater than that of the undoped NZSP, which displays a conductivity of 0.37 mS cm^−1^ at 25 °C. Moreover, the activation energy (*E*_a_) for Na^+^‐ion conductivity in 0.15Ca‐NZSP, as determined by the Arrhenius plots in the 5–65 °C range, is found to be 0.32 eV, compared to the *E*_a_ of 0.37 eV for the pristine NZSP (Figure S6, Supporting Information). It is, therefore, evident that Ca^2+^‐ion doping markedly augments the Na^+^‐ion transport within the NASICON structure. The enhanced conductivity can be comprehended in three aspects: 1) the substitution of low‐valence Ca^2+^‐ions for high‐valence Zr^4+^ ions leads to an increase in the Na^+^‐ion concentration within the lattice for charge compensation. Given that the Na1 site (NaO_6_ octahedral interstice) in the monoclinic Na_3_Zr_2_Si_2_PO_12_ is fully occupied, the increased Na^+^ ions primarily enhance the occupancy of other Na^+^‐ion sites, facilitating multisite Na^+^‐ion coordinated migration with a considerably lower barrier than singular migration,^[^ ^7^, ^41^, ^42^, ^43^ ^]^ thereby elevating the conductivity; 2) the larger ionic radii of Ca^2+^ (0.106 nm) compared to Zr^4+^ (0.072 nm) trigger cell expansion along the *a*‐axis and *c*‐axis directions (Figure S2, Supporting Information) and enlarge the Na^+^‐ion diffusion bottlenecks (triangles created by three O atoms from SiO_4_/PO_4_ tetrahedrons), promoting Na^+^‐ion diffusion within the lattice of Ca‐doped NZSP, and leading to a denser microstructure than the undoped NZSP;^[^ ^38^, ^42^, ^43^ ^]^ 3) the Ca^2+^‐ion dopant positively influences the densification sintering of these NZSP‐based solid electrolytes (refer to SEM images in Figure S3 in the Supporting Information), significantly enhancing the Na^+^‐ion transport across the grain boundaries.^[^ ^41^, ^42^ ^]^ However, excessive doping beyond the Ca^2+^‐ion solubility limit (*x* > 0.15) results in fewer vacancies for efficient Na^+^ migration^[^ ^41^, ^44^ ^]^ and introduces more insulating secondary phases^[^ ^37^ ^]^ (refer to XRD patterns in Figure 1a), which accounts for the decrease in conductivity with an increased doping ratio beyond 0.15.

**Figure 2:**
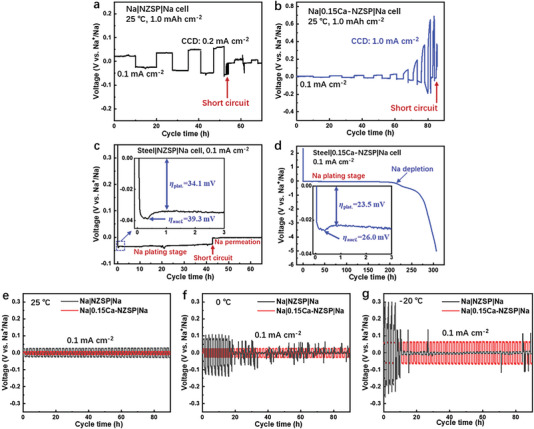
Critical current density (CCD) tests of the a) NZSP and b) 0.15Ca‐NZSP SEs measured at 25 °C. Discharge profile of the c) steel|NZSP|Na cell and d) steel|0.15Ca‐NZSP|Na cell under 0.1 mA cm^−2^. Galvanostatic cycling profile of the symmetrical Na|0.15Ca‐NZSP|Na cell with those of the Na|NZSP|Na cell as comparison at different temperatures: e) 25 °C, f) 0 °C, and g) −20 °C.

**Figure 3:**
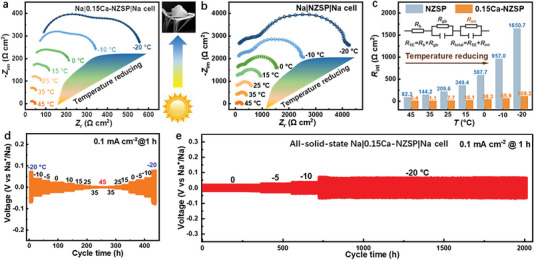
Temperature‐resolution Nyquist plots of a) the symmetrical Na|0.15Ca‐NZSP|Na cell and b) the Na|NZSP|Na cell in the temperature range from −20 to 45 °C. c) The calculated interfacial resistance (*R*_int_) at Na/0.15Ca‐NZSP interface with that of the pristine NZSP as comparison; inset: the equivalent circuit. d) Consecutive galvanostatic discharge/charge cycles of the Na|0.15Ca‐NZSP|Na cell from −20 to 45 °C and then back to −20 °C at 0.1 mA cm^−2^. e) Long‐term galvanostatic charge/charge cycles at 0.1 mA cm^−2^ with the temperature stepwise reduced from 0 to −20 °C.

A critical current density (CCD) is an important parameter of a SE representing the permitted up‐limit current density beyond which uncontrollable metal permeation across the SE and a short circuit finally occur. The 0.15Ca‐NZSP was elected as the SE in symmetric Na||Na cells for CCD testing, with NZSP serving as a reference. The CCD outcomes for NZSP and 0.15Ca‐NZSP, demonstrated in **Figure** **2**a,b, respectively, were assessed at 25 °C under a steady areal capacity of 1.0 mAh cm^−2^. The 0.15Ca‐NZSP showcased a CCD of 1.0 mA cm^−2^, fivefold greater than that of the NZSP (0.2 mA cm^−2^), indicating the superior capacity of 0.15Ca‐NZSP in facilitating high‐flux Na^+^‐ion transport, even at 25 °C. The escalating voltages noted in the CCD curve of 0.15Ca‐NZSP, starting from 0.5 mA cm^−2^, are credited to rapid Na‐metal exhaustion‐induced electrochemical polarization at the interface under high current densities. Galvanostatic discharge curves of the steel|NZSP|Na cell and the steel|0.15Ca‐NZSP|Na cell were collected to examine the Na‐metal plating behavior on the SE surface under 0.1 mA cm^−2^ at 25 °C. As depicted in Figure 2c,d, both curves contain a Na‐metal nucleation stage with a characteristic nucleation overpotential (*η*_nucl._) and a Na‐metal plating stage with a plating potential (*η*_plat._). The 0.15Ca‐NZSP bestows desirable Na‐metal plating kinetics with smaller *η*_nucl._ (26.0 mV) and *η*_plat._ (23.5 mV) compared to those of the NZSP cell (39.3 and 34.1 mV, respectively). Additionally, the 0.15Ca‐NZSP cell exhibits a stable Na plating voltage with an extraordinarily high areal capacity of 20.0 mAh cm^−2^ before Na depletion instigates increased polarization. Conversely, the NZSP cell only portrays a small areal capacity of 4.22 mAh cm^−2^, after which a short‐circuit transpires. These observations distinctly demonstrate that the synthesized 0.15Ca‐NZSP SE inherently possesses beneficial electrochemical kinetics attributes for high‐flux Na‐metal plating and stripping. Further, the symmetrical Na|NZSP|Na and Na|0.15Ca‐NZSP|Na cells were scrutinized for their galvanostatic charging/discharging cycles at various temperatures of 25, 0, and −20 °C under a constant current density of 0.1 mA cm^−2^. As depicted in Figure 2e, both NZSP and 0.15Ca‐NZSP demonstrate reversible Na‐metal plating/stripping cycles for 90 h at 25 °C. However, the 0.15Ca‐NZSP indicates a lower voltage of 8.7 mV versus Na^+^/Na, compared to the NZSP, which exhibits a higher voltage of 28.0 mV versus Na^+^/Na. Remarkably, the 0.15Ca‐NZSP is capable of maintaining long‐term stable Na‐metal plating/stripping cycles for more than 800 h, even under progressively increased current densities of up to 0.8 mA cm^−2^ at 25 °C. In stark contrast, NZSP maintains reversible Na‐metal plating/stripping cycles for only 30 h, when subjected to stepwise increased current density from 0.1 to 0.2 mA cm^−2^ at the same temperature (Figure S7, Supporting Information). As shown in Figure  2f and g, the Na|NZSP|Na cell exhibits large voltage fluctuations of more than 100 mV (0 °C) and 300 mV (−20 °C). During a cycling time of 20 h, Na^+^/Na experiences rapid short circuits, whereas the Na|0.15Ca‐NZSP|Na cell continues to sustain sturdy Na‐metal plating/stripping cycles with stable voltages of 24.0 mV (at 0 °C) and 62.6 mV (at −20 °C). These findings suggest that 0.15Ca‐NZSP considerably enhances the Na‐metal plating/stripping activities at the interface. It also indicates that 0.15Ca‐NZSP holds considerable promise for applications across a broad temperature range, demonstrating resilience even at severe temperatures as low as −20 °C. To elucidate the interfacial electrochemical process for temperature adaptation, TR‐EIS measurements of the symmetrical cells were performed across a temperature range from −20 to 45 °C. **Figure** **3**a,b displays the collected Nyquist plots for the Na|0.15Ca‐NZSP|Na cell and the Na|NZSP|Na cell, respectively. The temperature‐dependent Nyquist plots of the Na|0.15Ca‐NZSP|Na cell are depicted across a temperature scope of −20 to 45 °C. The interfacial resistance (*R*_int_) and total cell resistance (*R*_cell_) are calculated in accordance with an equivalent circuit, as illustrated in the inset of Figure 3c. This includes the corresponding *R*_int_ comparisons between the two symmetrical cells at various temperatures. It is observed that both the *R*_int_ and *R*_cell_ for NZSP are multiple times higher than those for 0.15Ca‐NZSP at each temperature within the −20 to 45 °C range. Specifically, the *R*_int_ values for the 0.15Ca‐NZSP with Na metal remain low at 0.4, 7.7, and 38.3 Ω cm^2^ at 45, 25, and 0 °C, respectively. Even at a frozen temperature of −20 °C, the *R*_int_ does not exceed 110 Ω cm^2^. In contrast, the *R*_int_ values for the NZSP with Na metal escalate dramatically from 82.3 Ω cm^2^ at 45 °C to 1650.7 Ω cm^2^ at −20 °C. In addition to *R*_int_, Figure S8 (Supporting Information) summarizes the *R*_cell_ values of symmetrical cells utilizing NZSP‐based solid electrolytes previously reported.^[^ ^26^, ^28^, ^30^, ^33^, ^45^, ^46^, ^47^, ^48^, ^49^, ^50^ ^]^ No symmetrical Na‐metal cells have been recorded to exhibit an *R*_cell_ of <100 Ω cm^2^ at 25 °C, let alone temperatures below 0 °C. Only those under additional external influences, such as ultrahigh pressure or ultrasound welding, have achieved an *R*_cell_ of ≈120 Ω cm^2^ at 25 °C. Remarkably, the 0.15Ca‐NZSP in our study not only offers the lowest *R*_cell_ of 76.8 Ω cm^2^ at 25 °C but also broadens the viable temperature range to be as low as −20 °C, at which the total *R*_cell_ is only 555.3 Ω cm^2^. Moreover, a galvanostatic Na‐metal plating/stripping cycling test of the Na|0.15Ca‐NZSP|Na cell at 0.1 mA cm^−2^ was performed under progressively varying temperatures from −20 to 45 °C and back to −20 °C. The cycling profile, shown in Figure 3d, exhibits consistent voltage steps in response to the temperature alterations. The resilient performance of the 0.15Ca‐NZSP is further emphasized when assessed in the symmetrical cell for extended Na‐metal plating/stripping cycles in the freezing temperature range from 0 to −20 °C. As illustrated in Figure 3e, the symmetrical Na|0.15Ca‐NZSP|Na cell delivers a stable voltage profile during the cycling time of 2000 h under 0.1 mA cm^−2^ and the progressively decreased temperature from 0 to −20 °C. Minor voltages of about 70 mV are consistently retained for more than 1250 h even under the extremely low temperature of −20 °C. Such a robust performance of the Na^+^‐ion SEs at extremely low temperatures, reaching down to −20 °C, has not been previously documented.

**Figure 4:**
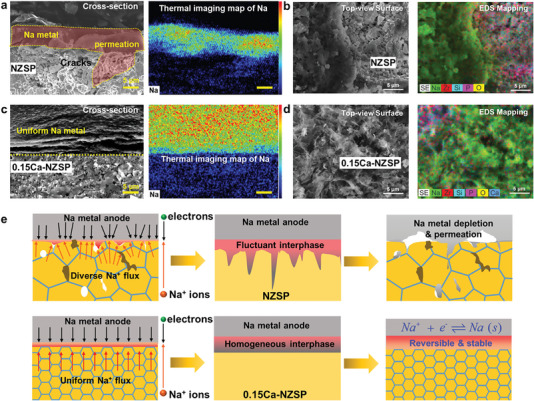
Cross‐sectional and top‐view SEM and elemental thermography mapping observation on the symmetrical cell after discharge/charge cycles for a,b) the NZSP and c,d) the 0.15Ca‐NZSP. e) Schematic of the mechanism for the homogeneous Na‐metal plating/stripping at the Na metal/0.15Ca‐NZSP interface with that of NZSP as comparison.

The cycled symmetrical cells were deconstructed for SEM examination in an Ar‐protected glovebox. As depicted in **Figure** **4**a, the cross‐sectional SEM image of the disassembled Na|NZSP|Na cell, along with its corresponding thermal imaging mapping of Na, is shown. It reveals the presence of cracks and the permeation of Na metal into the NZSP bulk, which indicates the electrochemical–mechanical failure of the Na|NZSP|Na cell. Figure 4b demonstrates obvious Na‐metal accumulation on the NZSP surface, suggesting coarse Na‐metal plating and subpar compatibility between Na and NZSP. Contrastingly, Figure 4c,d reveals that the disassembled Na|0.15Ca‐NZSP|Na cell exhibits a strong interfacial connection between Na metal and 0.15Ca‐NZSP with no evidence of Na permeation or dendrites. This suggests homogeneous Na‐metal plating/stripping at the contact interface. With reference to solid‐state thermodynamics and phenomenological transport theory,^[^ ^51^, ^52^, ^53^, ^54^ ^]^ a schematic mechanism considering the characteristics of NZSP and 0.15Ca‐NZSP is proposed in Figure 4e. The NZSP, possessing a loose microstructure and coarse grains, leads to broad and fluctuating potential differences at the contact interface with Na metal. This, in turn, prompts uneven interphase formation, ultimately driving distinctive Na‐metal plating and dendrite growth. Conversely, the 0.15Ca‐NZSP, characterized by a denser microstructure and superior grain conductivity, fosters uniform interface contact and even potential distribution at the Na metal/0.15Ca‐NZSP interface. A beneficial, conformal interphase layer is formed,^[^ ^55^ ^]^ stabilizing the Na‐metal anode and facilitating smooth Na‐metal plating/stripping with small interfacial resistance, even at low temperatures.

**Figure 5:**
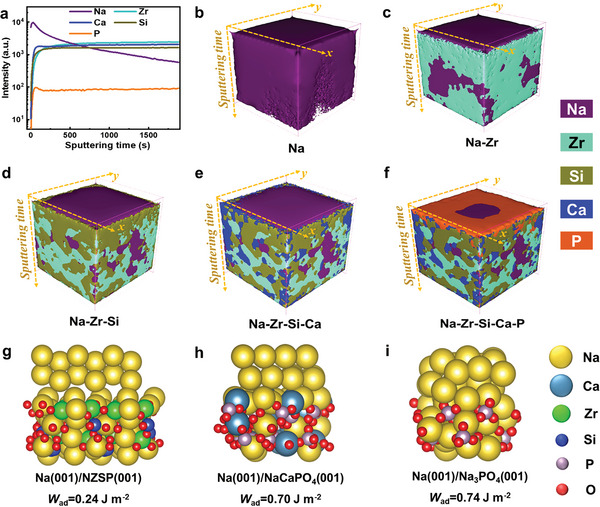
In‐depth analysis of the Na metal/0.15Ca‐NZSP interphase by time‐of‐flight secondary ion mass spectroscopy (ToF‐SIMS) and the work of adhesion (*W*_ad_) calculations using the Vienna ab initio simulation package (VASP). a) In‐depth profile of Na, Zr, Ca, Si, and P and the corresponding 3D distribution models of b) Na, c) Na–Zr, d) Na–Zr–Si, e) Na–Zr–Si–Ca, and f) Na–Zr–Si–Ca–P. The structures and corresponding *W*_ad_ values of g) Na (001)/NZSP (001), h) Na (001)/NaCaPO_4_ (001), and i) Na (001)/Na_3_PO_4_ (001).

The interphase resulting from cycling and its impact on interfacial performance were clarified using time of flight secondary ion mass spectroscopy (ToF‐SIMS) and in‐depth XPS analysis across the Na metal/0.15Ca‐NZSP interface. For these evaluations, the sample was taken from the Na|0.15Ca‐NZSP|Na symmetrical cell after cycling. Specialized transfer vessels, which facilitate the direct transfer of samples from the argon‐filled glovebox to the XPS and ToF‐SIMS vacuum chambers without exposing the sample to air, were utilized. ToF‐SIMS measurements were conducted using an Ar^+^ beam (3 kV 100 nA) on a sputtering area of 400 µm × 400 µm while setting the analysis area to be 100 µm × 100 µm. **Figure** **5**a–f presents the in‐depth profile of Na, Zr, Ca, Si, and P species along with derived 3D distribution models of Na and overlays of Na–Zr, Na–Zr–Si, Na–Zr–Si–Ca, and Na–Zr–Si–Ca–P for enhanced observation of the interphase. The results show a distinct and thin Na layer in the sputtering time range of 0–30 s (≈5 nm as calculated from the calibrated sputtering speed of 0.16–0.17 nm s^−1^). Beneath this layer, in the sputtering time range of 30–150 s (roughly 15 nm), Ca and P species appear and aggregate with Na, suggesting a thin interphase layer dominated by Na–Ca–P–O species. Upon 150 s of sputtering, the intensity of Zr, Si, Ca, and P species tends toward a constant value, and the Na content gradually diminishes until the sputtering time reaches up to 1900 s (≈317 nm), indicative of a characteristic of the 0.15Ca‐NZSP bulk.

Figure S9 (Supporting Information) presents the in‐depth XPS spectra of Na 1s, Ca 2p, Zr 3d, Si 2p, P 2p, and O 1s with the C 1s calibration for the surfaces after 0–5 cycles of Ar^+^‐ion etching (each etching lasting for 50 s). It demonstrates that the disassembled 0.15Ca‐NZSP has a Na‐rich surface featuring weak‐binding Ca, Zr, Si, and P species in small quantities, and a Na_2_CO_3_ component (originating from the reaction between the deposited Na metal and some residual CO_2_). The weak‐binding Ca, Zr, Si, and P species are associated with the chemical reduction reaction of 0.15Ca‐NZSP by Na metal at the interface. After removing the first layer by etching for 50 s (≈10 nm), the binding energies of Ca, Zr, Si, and P species increase to their typical oxide states in the highest valences. This suggests that the chemical reduction of 0.15Ca‐NZSP by Na metal occurs solely at the contact interface without extending into the bulk.^[^ ^23^, ^45^ ^]^ These findings align with the ToF‐SIMS analysis and further, confirm the presence of a stable interphase layer at the interface to sustain homogeneous Na‐metal plating/stripping throughout discharge/charge cycles.

Building upon the above analysis, we propose that a dynamically stable Na_3−_ *_x_* Ca*_x_* PO_4_‐dominant interphase contributes to the superior interfacial performance observed. In order to unravel the microscopic mechanisms by which such an interphase improves interfacial contact, we conducted spin‐polarized density functional theory (DFT) computations^[^ ^56^, ^57^ ^]^ employing the Vienna ab initio simulation package (VASP) based on plane‐wave basis sets and the projector augmented‐wave method.^[^ ^58^, ^59^ ^]^ We selected slabs of NZSP (001) and two characteristic compounds of NaCaPO_4_ (001) and Na_3_PO_4_ (001), representing the Na_3−_ *_x_* Ca*_x_* PO_4_ interphase, to couple with Na (001) and construct three typical interface models. The work of adhesion (*W*_ad_) is defined as follows:^[^ ^24^, ^26^ ^]^ *W*_ad_ = (*E*_Na+_ *E*_slab_ − *E*_Na/slab_)/*A*, where *E*_Na_, *E*_Na/slab_, and *E*_slab_ signify the total energies of the Na (001) surface, Na (001)/slab interfaces, and the slabs, respectively, and *A* represents the interfacial area. As demonstrated in Figure 5g–i, the *W*_ad_ values of the three interface models are 0.24, 0.70, and 0.74 J m^−2^, respectively. The tripled *W*_ad_ for the NaCaPO_4_ and Na_3_PO_4_ slabs compared to that for the NZSP slab suggests that these phosphate‐based interphases enhance interfacial contact with Na metal. The outstanding interfacial performance of 0.15Ca‐NZSP can be attributed to its dense microstructure and advantageous interface chemistry upon contact with Na metal.

**Figure 6:**
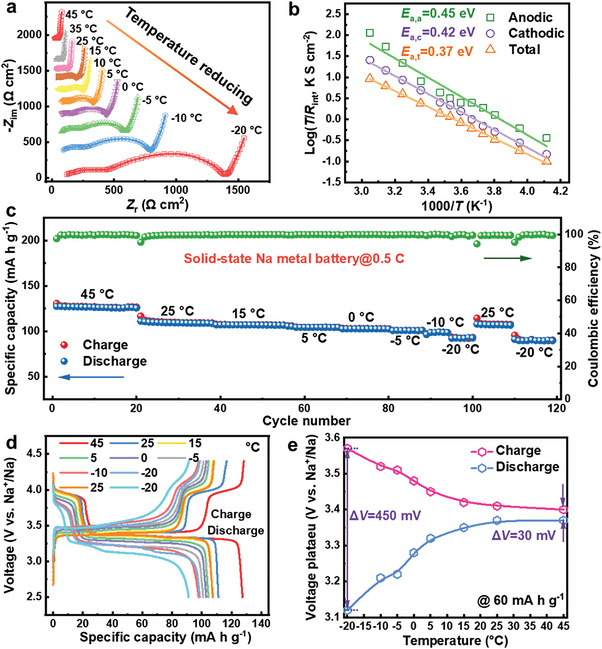
Temperature‐dependent electrochemical performance of the solid‐state NVAP|0.15Ca‐NZSP|Na battery. a) Nyquist plots and b) Arrhenius plots of the conductivity in the range from −20 to 45 °C. c) Cycling performance and d) galvanostatic profile at a charge/discharge rate of 0.5 C measured under the stepwise changed temperature from 45 to −20 °C. e) The charge/discharge voltages versus temperature collected at the capacity of 60 mA h g^−1^.

Subsequently, we prepared a composite cathode consisting of a NASICON‐type Na_3_V_1.5_Al_0.5_(PO_4_)_3_ (NVAP) cathode material and a plastic‐crystal electrolyte (PCE) of NaClO_4_‐succinonitrile^[^ ^28^, ^60^ ^]^ along with a polyvinylidene fluoride (PVDF) binder, and acetylene black was prepared for full battery measurement. NVAP was synthesized via a sol–gel method, as described earlier.^[^ ^61^ ^]^ The XRD pattern, TEM, and high‐resolution TEM images in Figure S10a–d (Supporting Information) confirm the rhombohedral structure of NVAP in an *R*‐3*c* space group, which is analogous to that of Na_3_V_2_(PO_4_)_3_ (NVP). A high‐angle annular dark field scanning transmission electron microscope (HAADF‐STEM) was employed to carry out the element mapping in NVAP. The homogeneous distribution of Na, V, Al, P, and O in the carbon‐coated NVAP particles is demonstrated in Figure S10e (Supporting Information). A 2032‐typed SSNMB in the configuration of NVAP‐PCE|0.15Ca‐NZSP|Na was assembled inside an Ar‐protected glovebox. **Figure** **6**a illustrates the TR‐EIS Nyquist plots of the NVAP‐PCE|0.15Ca‐NZSP|Na battery at different temperatures in the range from −20 to 45 °C. An equivalent circuit consisting of the resistance from the solid electrolyte (*R*_SE_), the anode interface (*R*_anode_), and the cathode interface (*R*_cathode_) is illustrated in Figure S11 (inset) (Supporting Information) along with the as‐calculated resistances versus the temperature. It is shown that *R*_SE_ dominantly contributes to the total resistance (*R*_total_) of the battery at the high‐temperature end, while the interfacial resistances, i.e., *R*_anode_ and *R*_cathode_ decide the battery resistance at the low‐temperature end. The lowest *R*_total_ of the battery is 37 Ω cm^2^ at 55 °C, while the highest *R*_total_ is 1409 Ω cm^2^ obtained at −20 °C. Typically, the *R*_total_ of the solid battery at room temperature is only 125 Ω cm^2^ that is close to that using the liquid electrolyte.^[^ ^61^ ^]^ Besides, a NVAP‐PCE|NZSP|Na battery was assembled for comparison, and it showed much larger *R*_total_ values, specifically 8000 Ω cm^2^ at −20 °C (Figure S12a, Supporting Information). Subsequently, the activation energy (*E*_a_) for charge transfer at the anodic and cathodic interfaces along with the total battery is calculated according to the Arrhenius equation^[^ ^49^ ^]^

(1) $$\frac{1}{R}=\frac{A}{T}\exp\left(-\frac{E_{a}}{k_{B}T}\right)$$

In the equation, *R* denotes the area‐specific resistance, *T* represents temperature, *k*_B_ stands for the Boltzmann constant, *A* is the pre‐exponential factor, and *E*_a_ signifies the activation energy. Figure 6b illustrates the linearly fitted Arrhenius plots for the anodic, cathodic, and total charge‐transfer processes. The corresponding activation energies for the NVAP‐PCE|0.15Ca‐NZSP|Na battery are found to be 0.45, 0.42, and 0.37 eV, respectively. The low *E*_a_ for charge transfer signifies conducive electrochemical reaction kinetics for effective Na^+^‐ion storage, even at a reduced temperature.

Figure 6c depicts the galvanostatic cycling performance of the SSNMB at 0.5 C (1 C = 124.7 mA g^−1^) under progressively decreased temperature from 45 to −20 °C, followed by resetting the temperature to 25 °C and further down to −20 °C. In the initial cycle at 45 °C, the battery provides a discharge capacity of 127.3 mA h g^−1^ with the first Coulombic efficiency at 97.4%. As the temperature drops to 25 °C, there is a decline in capacity to 111.2 mA h g^−1^ accompanied by a decreased Coulombic efficiency of 95.2%, reflecting the decelerated interfacial kinetics. As the temperature fluctuates between 15 and −5 °C, the charge/discharge capacity experiences a marginal decline, maintaining a highly reversible capacity of 102.0 mA h g^−1^ even at the significantly colder temperature of −5 °C. Lowering the temperature further to −10 and −20 °C triggers noticeable capacity fluctuations, yet the capacity rebounds to 108.3 mA h g^−1^ as the battery warms to 25 °C. Following another cooling cycle to −20 °C, the battery sustains a stable capacity of 90.0 mA h g^−1^ after the 120th cycle, indicating exceptional low‐temperature cycling performance. Figure 6d offers a glance at the selected voltage profile at each temperature. Notable charge/discharge voltage plateaus are observed, attributable to the two‐electron transfer processes of V^3+^/V^4+^ and V^4+^/V^5+^ redox reactions originating from the Na^+^‐ion extraction/insertion events of the NVAP cathode.^[^ ^61^ ^]^ Figure 6e illustrates the charge and discharge voltages at a capacity of 60.0 mA h g^−1^, shedding light on the polarization as the temperature varies. A marginal increase in the polarization voltage (Δ*V*) is noticed as the temperature reduces from 45 °C (Δ*V* = 30 mV) to 15 °C (Δ*V* = 70 mV). Subsequently, Δ*V* escalates swiftly, reaching 450 mV at −20 °C. This rise in Δ*V* is attributable to the slowed charge‐transfer process at low temperatures, a phenomenon in line with the EIS results presented in Figure 6a.

**Figure 7:**
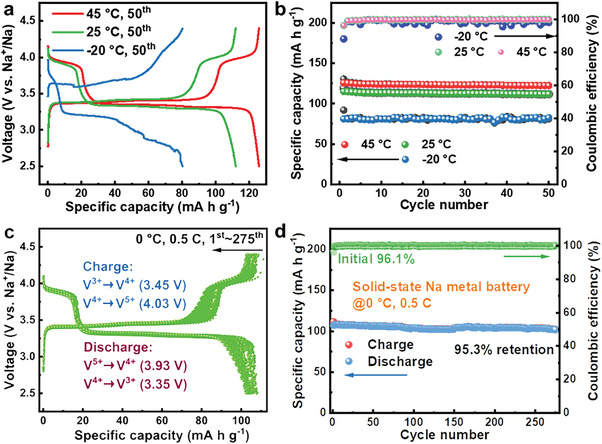
Galvanostatic cycling of the solid‐state Na_3_V_1.5_Al_0.5_(PO_4_)_3_|0.15Ca‐NZSP|Na‐metal battery at constant temperatures. a) The selected 50th charge/discharge cycling profile and b) the cycling performance tested at 45, 25, and −20 °C. c) The 1st to 275th charge/discharge cycling profile and d) the cycling performance at 0.5 C rate and 0 °C.

The temperature adaptability was further assessed through galvanostatic charge/discharge cycling at constant temperatures. **Figure** **7**a presents the voltage profile of the 50th charge/discharge cycle at 45, 25, and −20 °C, respectively. Post the 50th cycle, the SSNMB values evaluated at 25 and 45 °C both exhibit smooth charge/discharge curves with two consistent discharge voltage plateaus at 3.94 and 3.35 V, comparable to the scenario with a liquid electrolyte.^[^ ^61^ ^]^ The initial discharge capacities are observed to be 115.0 mA h g^−1^ at 25 °C and 126 mA h g^−1^ at 45 °C with initial Coulombic efficiencies of 96.3% and 96.4%, respectively. After 50 cycles, the capacity retentions at 25 and 45 °C are 97.1% and 97.2%, respectively. In contrast, when tested at −20 °C, the battery shows a reduced capacity of around 81.2 mA h g^−1^ and a lower initial Coulombic efficiency of 88.2%, due to significant electrochemical polarization. Nevertheless, the SSNMB at −20 °C exhibits an impressive retention of nearly 100% over 50 cycles and an average Coulombic efficiency of 98.5% (Figure 7b). In stark contrast, the NVAP‐PCE|NZSP|Na battery, with a high battery resistance, demonstrates inferior cycling performance, with rapid battery failure within just ten cycles at −20 °C (Figure S12b–d, Supporting Information). Specifically, galvanostatic charge/discharge cycles at 0 °C were conducted to further highlight the low‐temperature performance of the solid‐state NVAP‐PCE|0.15Ca‐NZSP|Na‐metal battery. Figure 7c displays the voltage profile for 275 charge/discharge cycles at 0.5 C and 0 °C. The overlapping voltage plateaus are clearly evident, and the charge/discharge curves exhibit minimal capacity decay even after the 275th cycle. The cycling performance, as shown in Figure 7d, indicates that the battery delivers a highly reversible capacity of 107.1 mA h g^−1^, accompanied by an initial Coulombic efficiency of 96.1% and a capacity retention of 95.3% after the 275th cycle. Moreover, the performance of the solid‐state NVAP‐PCE|0.15Ca‐NZSP|Na battery surpasses those of most NZSP‐based solid‐state batteries documented in previous literature (Table S1, Supporting Information).^[^ ^20^, ^24^, ^27^, ^29^, ^30^, ^31^, ^32^, ^45^, ^46^, ^47^, ^50^, ^62^ ^]^ The robust wide‐temperature performance of the NVAP‐PCE|0.15Ca‐NZSP|Na battery can be credited to the steady Na‐metal plating/stripping behavior at the anodic interface and the excellent low‐temperature properties of the NVAP cathode material.^[^ ^61^ ^]^

## 3 Conclusion

To summarize, the temperature adaptability of a solid‐state Na‐metal battery is exhaustively studied across a broad temperature spectrum ranging from −20 to 45 °C. Primarily, an optimized Ca^2+^‐doped Na_3.3_Zr_1.85_Ca_0.15_Si_2_PO_12_ (abbreviated as 0.15Ca‐NZSP) solid electrolyte is fabricated via a solid‐state reaction technique in a pure oxygen environment. The 0.15Ca‐NZSP, characterized by a dense microstructure and a favorable Na‐compatible surface, boasts a high room‐temperature conductivity of 1.59 mS cm^−1^ and exhibits superior performance for Na‐metal plating/stripping. Remarkably, the all‐solid‐state symmetrical Na|0.15Ca‐NZSP|Na cell imparts significantly reduced interfacial resistances of 0.4 Ω cm^2^ at 45 °C and 7.7 Ω cm^2^ at 25 °C. Even at a low temperature of −20 °C, the interfacial resistance remains less than 110 Ω cm^2^, suggesting optimal electrochemical reaction kinetics. Furthermore, highly reversible Na‐metal plating/stripping cycles at 0.1 mA cm^−2^ are achieved under a gradually reduced temperature from 45 to −20 °C. Additionally, sustained uniform Na‐metal plating/stripping cycles are accomplished for 2000 h in the frozen temperature region from 0 to −20 °C. ToF‐SIMS and XPS analysis, along with the DFT calculations, illustrate that a uniform Na_3−_ *_x_* Ca*_x_* PO_4_ interphase layer formed between Na metal and 0.15Ca‐NZSP contributes to exceptional interfacial performance. When paired with a NASICON‐type Na_3_V_1.5_Al_0.5_(PO_4_)_3_‐based composite cathode, the solid‐state Na‐metal battery is assembled and displays impressive cycling performance at −20 °C with a reversible capacity of 80 mA h g^−1^ and nearly 100% retention over 50 charge/discharge cycles. Additionally, a high capacity of 108 mAh g^−1^ at 0 °C is delivered, maintaining over 95% retention after the 275th cycle. This study represents a significant advancement in the field of inorganic solid electrolyte‐based Na‐metal batteries and lays the foundation for the development of all‐season applicable solid‐state power sources.

## Conflict of Interest

The authors declare no conflict of interest.

## Supporting information

Supporting Information

## Data Availability

Research data are not shared.
