# Supplementary material for: Rechargeable Solid‐State Na‐Metal Battery Operating at −20 °C
Source: Adv Sci (Weinh). 2023 Jul 23;10(27):2302774. doi: 10.1002/advs.202302774 (PMC10520632; doi:10.1002/advs.202302774)
Supplement: Supplementary file 1 — Supporting Information [file ADVS-10-2302774-s001.pdf]

ADVANCED SCIENCE
Open Access

Supporting Information

for *Adv. Sci.*, DOI 10.1002/advs.202302774

Rechargeable Solid-State Na-Metal Battery Operating at −20 °C

*Haibo Jin, Xiong Xiao, Lai Chen, Qing Ni, Chen Sun, Runqing Miao, Jingbo Li, Yuefeng Su and Chengzhi Wang**

1. Experimental Section

Synthesis of the solid electrolyte: A solid-state reaction method was adopted to synthesize Ca^{2+}-ion doped $Na_3Zr_2Si_2PO_{12}$ with a chemical formula of $Na_{3+2x}Zr_{2-x}Ca_xSi_2PO_{12}$ (denoted as *x*Ca-NZSP, *x*=0, 0.05, 0.10, 0.15, 0.20, 0.25, 0.30) using anhydrous Na_2CO_3, $ZrO(NO_3)_2$, CaC_2O_4, SiO_2, NaH_2PO_4 as raw materials. The raw mixture was pre-sintered at 1000 °C for 12 h. Then, the pre-sintered powder was pressed into discs of around 10 mm in diameter with the assistance of a polyvinyl butyral (PVB) binder which was subsequently pyrolyzed at 650 °C for 3 h in air. Final sintering was conducted at 1200 °C for 12 h with a ramp rate of 5 °C min^{-1}. All the sintering processes were performed in a pure-O_2 atmosphere within a tube furnace.

Synthesis of the cathode material: A NASICON-type $Na_3V_{1.5}Al_{0.5}(PO_4)_3$ (NVAP) cathode material was synthesized via a sol-gel method according to the literature[1]. Typically, 3 mmol of CH_3COONa, 0.5 mmol $Al(NO_3)_3{\cdot}9H_2O$, 1.5 mmol NH_4VO_3, 3 mmol $NH_4H_2PO_4$ and 4 mmol citric acid ($C_6H_8O_7{\cdot}H_2O$) were dissolved into 20 mL of deionized water. The solution was magnetically stirred for 3 h at 50 °C to form a homogeneous solution. The mixture was then dried in an oven at 120 °C for 12 h to get a gel. The product was ground into powder and heated at 800 °C for 10 h with a ramp rate of 5 °C min^{-1} in a tube furnace under Ar atmosphere.

Synthesis of the plastic-crystal electrolyte: The plastic-crystal electrolyte (PCE) was prepared as an ionic conductive additive of the cathode[2-3]. Typically, 0.1 mmol of $NaClO_4$ was dissolved in 2 mmol of succinonitrile at 65 °C to form a clear solution. After cooling to room temperature, a yellow and sticky plastic-crystal electrolyte was obtained.

Characterization: X-ray diffraction (XRD, SmartLab, Rigaku Co; Cu-*K*α radiation) along with the Rietveld refinement method was used to analyze the phase structure. Field emission scanning electron microscope (FESEM, Hitachi Regulus8230) equipped with an energy dispersive spectrometer (EDS) and field emission transmittance electron microscope (FETEM, FEI Tecnai F30) were adopted for microstructural and composition characterization. Surface chemical states were analysed by X-ray photoelectron spectroscopy (XPS, Thermo Scientific *K*-Alpha). For XPS characterization, the Ar-protected sample injection was performed using a transition chamber through an Ar-filled glove box. The apparent densities of the solid electrolytes were determinded by

an Archimedes drainage method. In-depth XPS analysis of the electrochemical reaction interface was performed through Ar^+-ion etching, where the spectra were collected every etching time of 50 s (corresponding to a 10-nm etching thickness). Time of flight secondary ion mass spectroscopy (ToF-SIMS, PHI nano ToF II, ULVAC-PHI) was used to perform the surface in-depth analysis, where an Ar^+ beam (3 kV 100 nA) was used to conduct the sputter etching. The analysis area was 100 μm×100 μm within the sputtering area of 400 μm×400 μm. A special transfer vessel, which can directly transfer the sample from the glovebox to the vacuum chamber of ToF-SIMS, was used during sample transfer without being exposed to ambient air.

Electrochemical test: Symmetrical Na||Na cells were assembled in an Ar-filled glovebox by directly placing Na metal foils on both sides of the solid electrolyte disc. Galvanostatic cycling of the symmetrical cells was conducted on a multi-channel battery test system. A thermostatic refrigeration/heating system (-40-50 °C) was used to control the test temperature. CR2032-typed solid-state Na metal batteries were assembled by using NVAP-PCE composite as the cathode, Na foil as the anode, *x*Ca-NZSP as the solid electrolyte. The NVAP-PCE composite cathode was fabricated by mixing NVAP powder, carbon black, polyvinylidene fluoride (PVDF) and the PCE at a weight ratio of 60: 5: 10: 25 in a mortar with N-methyl-2-pyrrodione (NMP) as a solvent. The obtained slurry was casted on the current collector of aluminum foil. After drying at 30 °C for 3 days under vacuum. Then the coated Al foil was punched into discs of 8 mm in diameter with the active mass loading of 1.2−1.5 mg cm^{-2}. Teperature-resolution electrochemical impedance spectroscopy (TR-EIS) analysis was conducted in the frequency range of 0.1-1 MHz with an amplitude of 5 mV on a CHI660E electrochemical workstation to reveal the charge transfer at the interfaces.

2. DFT calculations

The spin-polarized density functional theory (DFT) calculations[4-5] were performed using the Vienna ab initio simulation package (VASP) based on the plane-wave basis sets with the projector augmented-wave method[6-7]. The exchange-correlation potential was treated via a generalized gradient approximation (GGA) with the Perdew-Burke-Ernzerhof (PBE) parametrization[8]. The van der Waals correction of Grimme's DFT-D3 model was adopted as well[9]. We constructed Na (001)/Na_3PO_4 (001), Na (001)/$NaCaPO_4$ (001) and Na (001)/$Na_3Zr_2Si_2PO_{12}$ (001) models, where a

vacuum region of about 15 Å was applied to avoid the interaction between adjacent images. The energy cutoff was set to be 520 eV. The Brillouin-zone integration was sampled with a Γ-centered Monkhorst-Pack mesh[10] of 2 × 2 × 1. The structures were fully relaxed until the maximum force on each atom was less than 0.01 eV/Å, and the energy convergent standard was 10^{-6} eV. The work of adhesion (W_{ad}) can be defined as: $W_{ad}=(E_{Na}+E_{slab}-E_{Na/slab})/A$, where E_{Na}, $E_{Na/slab}$ and E_{slab} represent the total energies of the Na (001) surface, Na (001)/slab interfaces and slabs [slab= Na_3PO_4 (001), $NaCaPO_4$ (001), $Na_3Zr_2Si_2PO_{12}$ (001)], respectively. A is the interfacial area.

3. Additional Figures

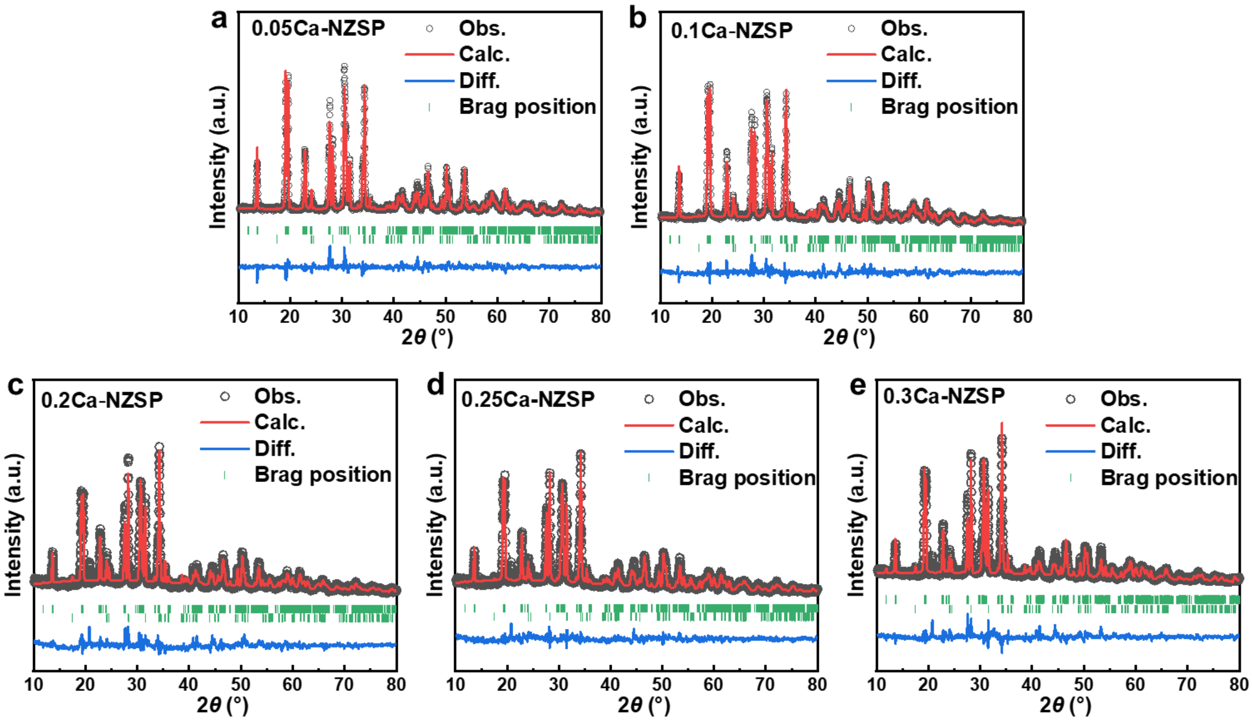

Figure S1. Rietveld refinement XRD results of (a) 0.05Ca-NZSP, (b) 0.1Ca-NZSP, (c) 0.2Ca-NZSP, (d) 0.25Ca-NZSP, (e) 0.3Ca-NZSP.

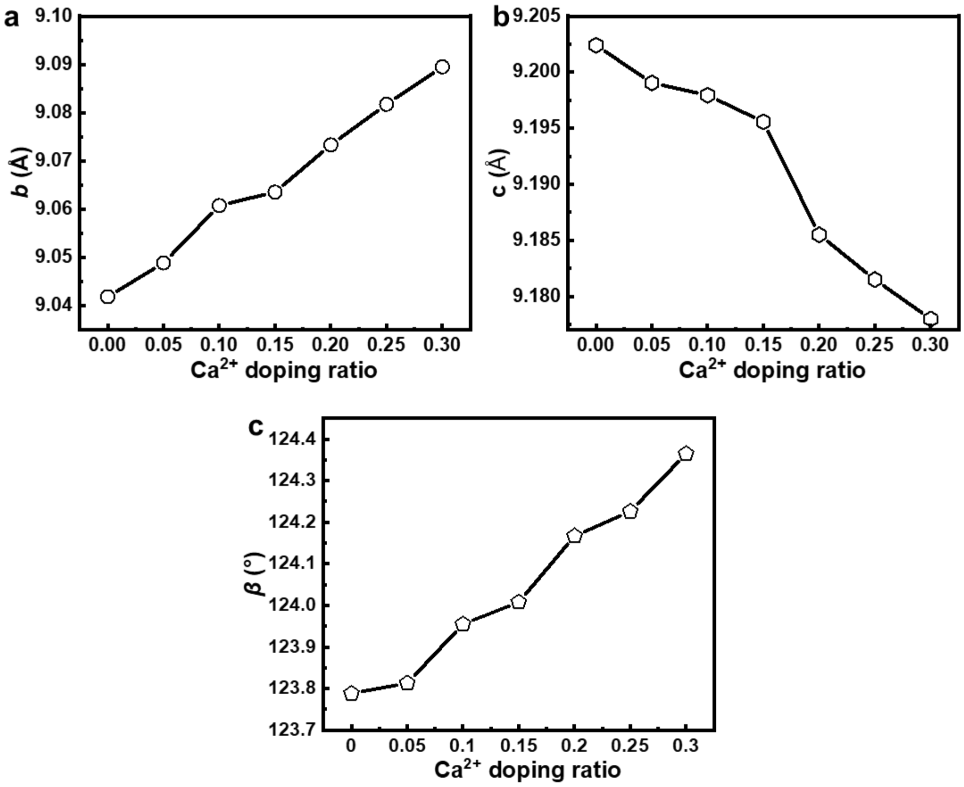

Figure S2. Relationship of the refined lattice parameters against the Ca^{2+}-ion doping ratio: (a) *b* (Å); (b) *c* (Å); (c) *β* (°).

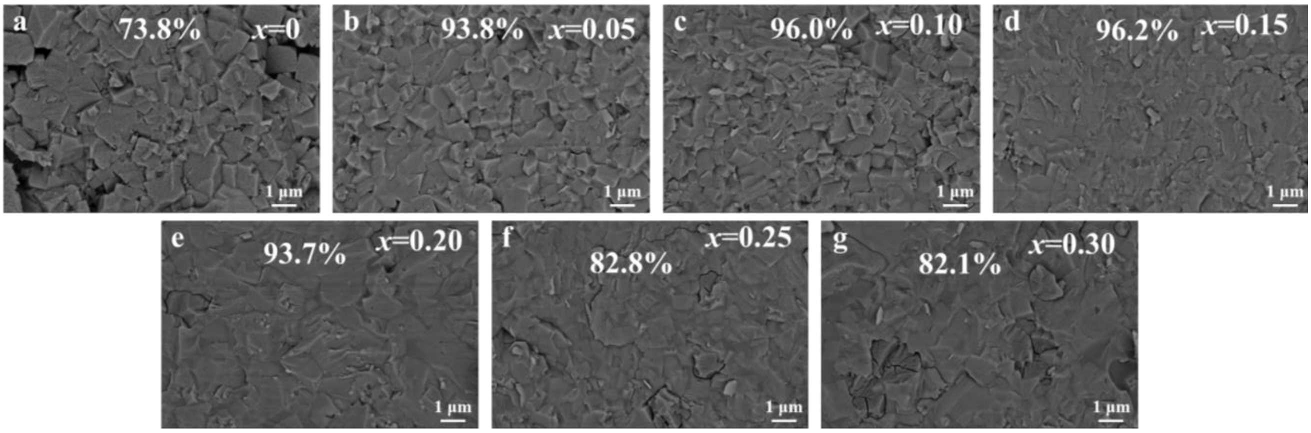

Figure S3. Cross-sectional SEM images of the *x*Ca-NZSP samples with the corresponding relative density determined by an Archimedes drainage method. (a) x=0; (b) x=0.05; (c) x=0.10; (d) x=0.15; (e) x=0.20; (f) x=0.25; (g) x=0.30.

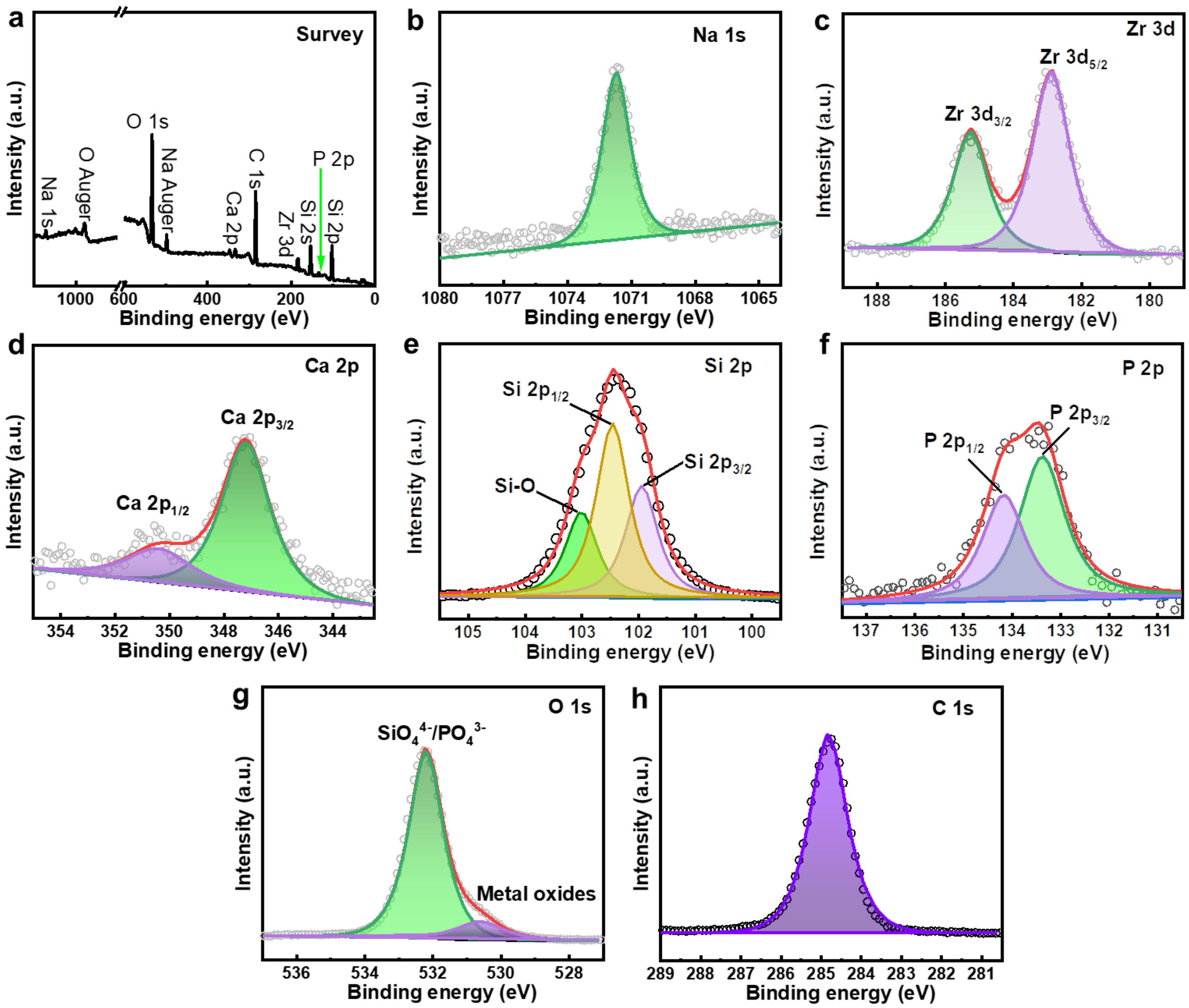

Figure S4. XPS spectra of the 0.15Ca-NZSP. (a) Survey spectrum, high-resolution spectra of (b) Na 1s, (c) Zr 3d, (d) Ca 2p, (e) Si 2p, (f) P 2p, (g) O 1s and (h) C 1s.

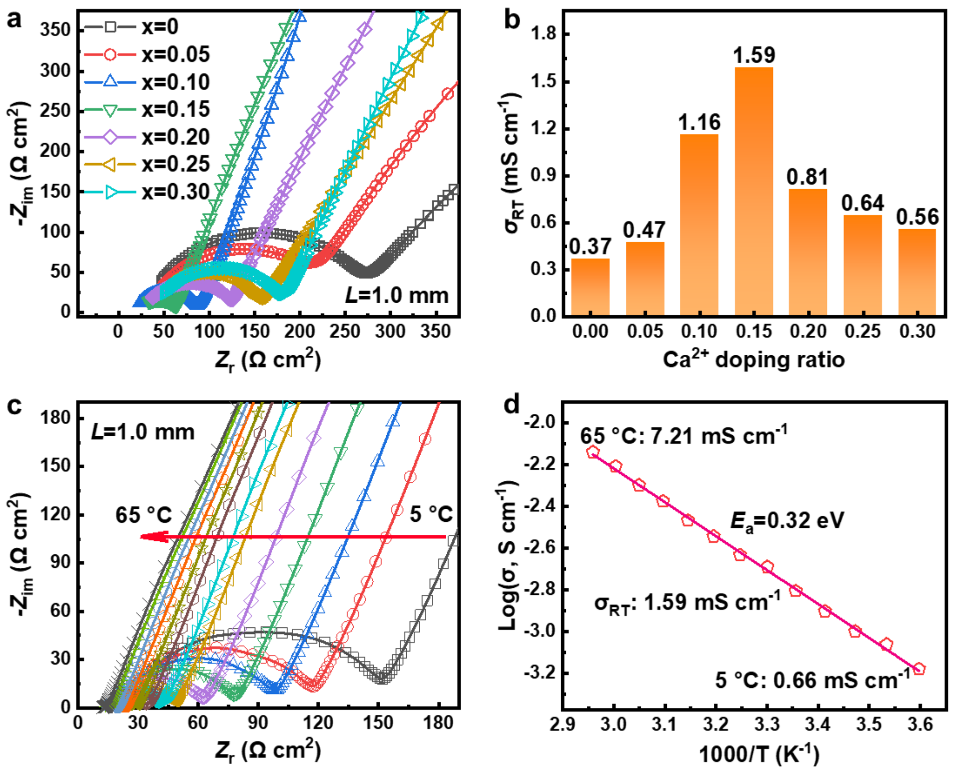

Figure S5. (a) Nyquist plots of *x*Ca-NZSP discs measured at 25 °C and (b) the corresponding room-temperature conductivity σ_{RT}. (c) Temperature-dependent Nyquist plots of 0.15Ca-NZSP discs measured at various temperatures from 5 to 65 °C. (d) Linear-fitted Arrhenius plot of the conductivity of 0.15Ca-NZSP.

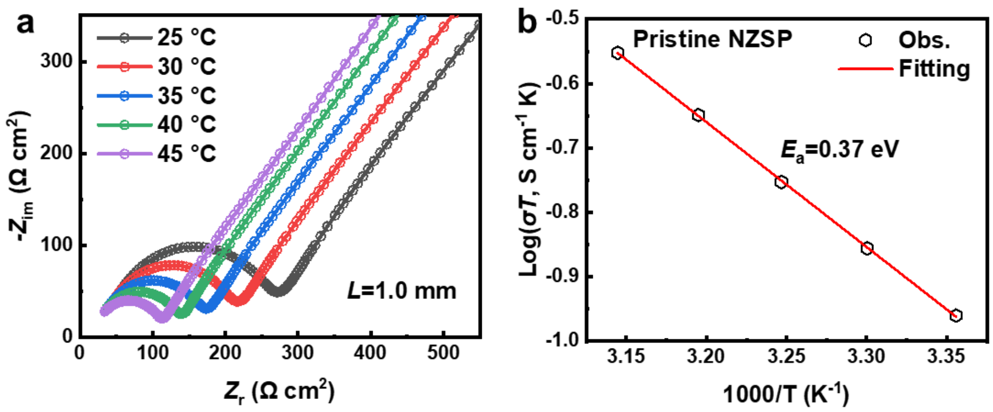

Figure S6. (a) Temperature-dependent Nyquist plots and (b) the corresponding Linear-fitted Arrhenius plot of the conductivity of NZSP measured in the temperature range of 25-45 °C.

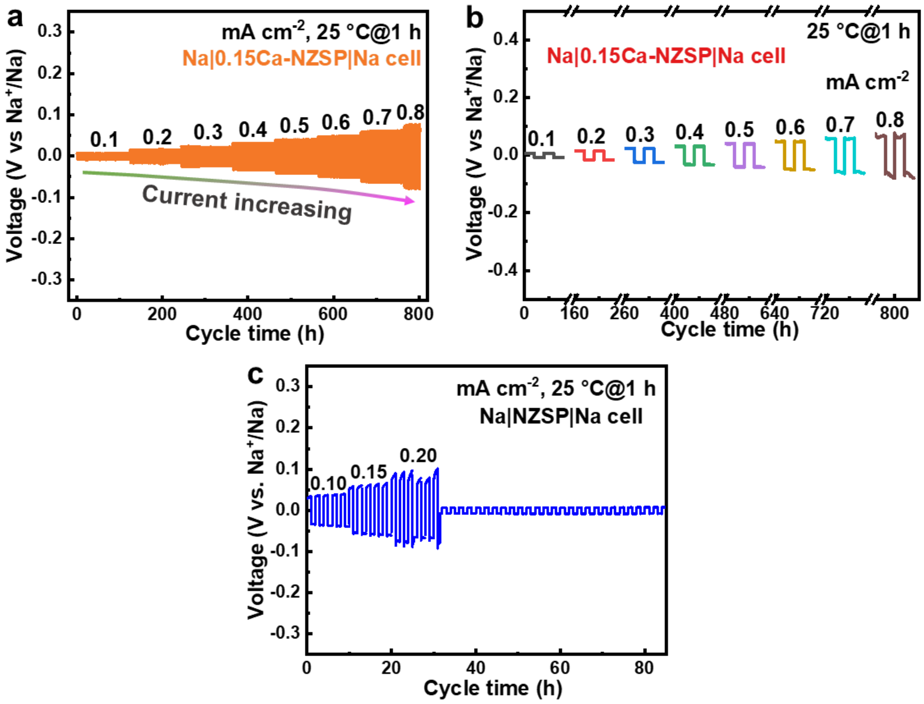

Figure S7. Galvanostatic charge/discharge cycling profile of (a) the symmetrical Na|0.15Ca-NZSP|Na cell under stepwise increased current densities from 0.1 to 0.8 mA cm^{-2} and (b) the magnified curves at selected cycling time. (c) Galvanostatic charge/discharge cycling profile of (a) the symmetrical Na|NZSP|Na cell under stepwise increased current densities from 0.10 to 0.20 mA cm^{-2} where a short circuit occurs.

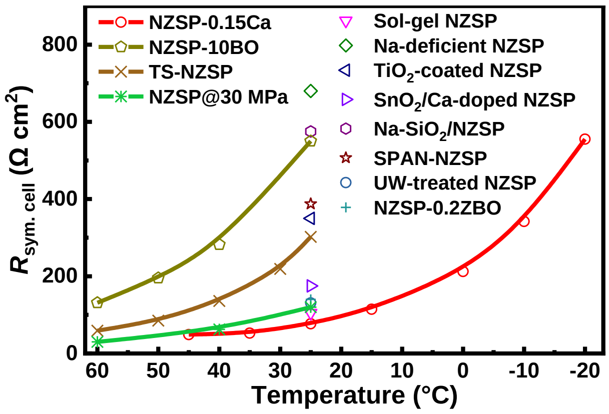

Figure S8. The symmetrical cell resistance ($R_{sym.\mathrm{cell}}$) for the 0.15Ca-NZSP compared with other NZSP-based solid electrolytes[3, 11-19] in the all-season temperature region.

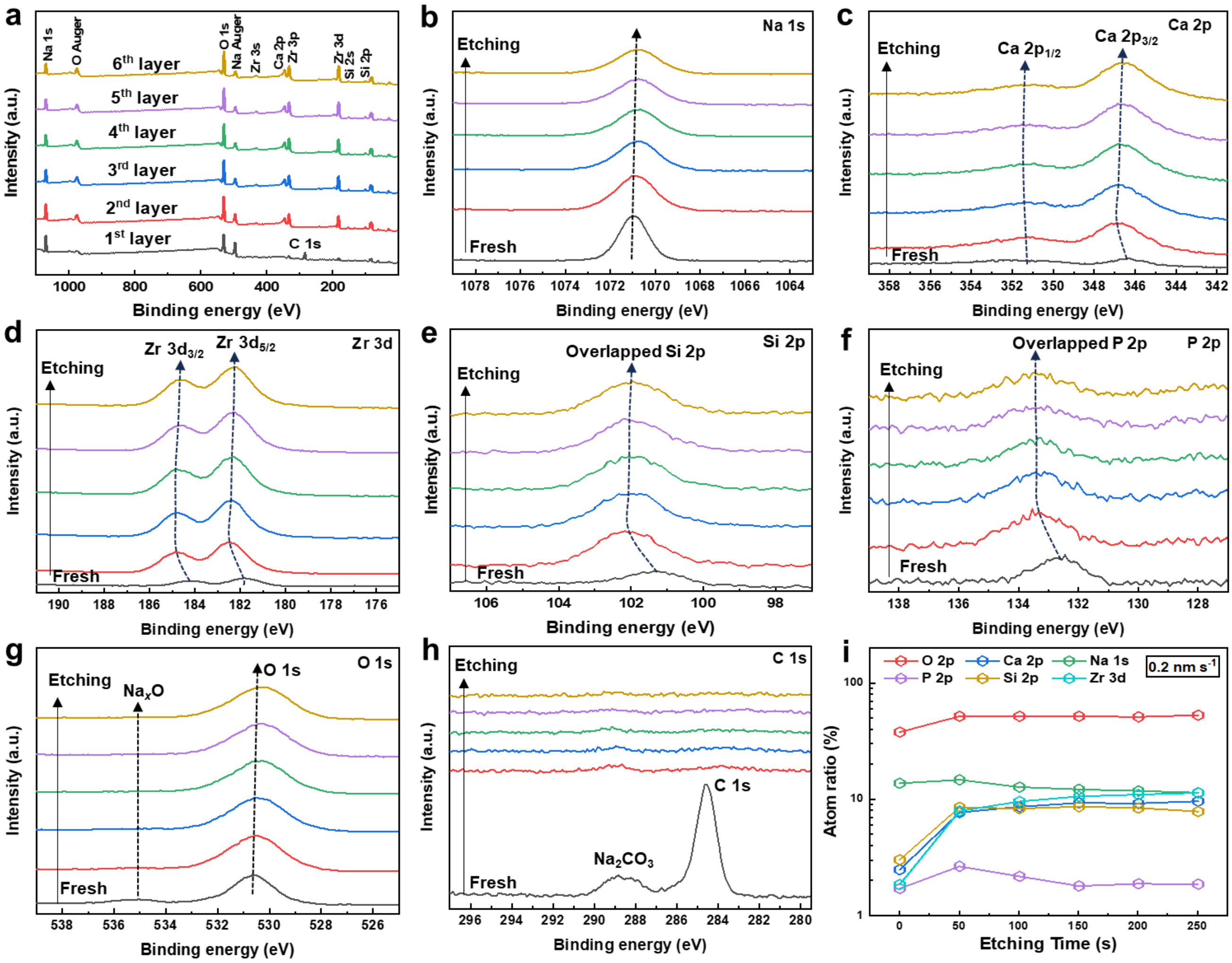

Figure S9. In-depth XPS spectra of the 0.15Ca-NZSP disassembled from the Na||Na symmetrical cell after cycling: (a) Survey, (b) Na 1s, (c) Ca 2p, (d) Zr 3d, (e) Si 2p, (f) P 2p, (g) O 1s, (h) C 1s and (i) the corresponding atom ratios versus the etching time. Each etching time is 50 s corresponding to an etching depth of about 10 nm.

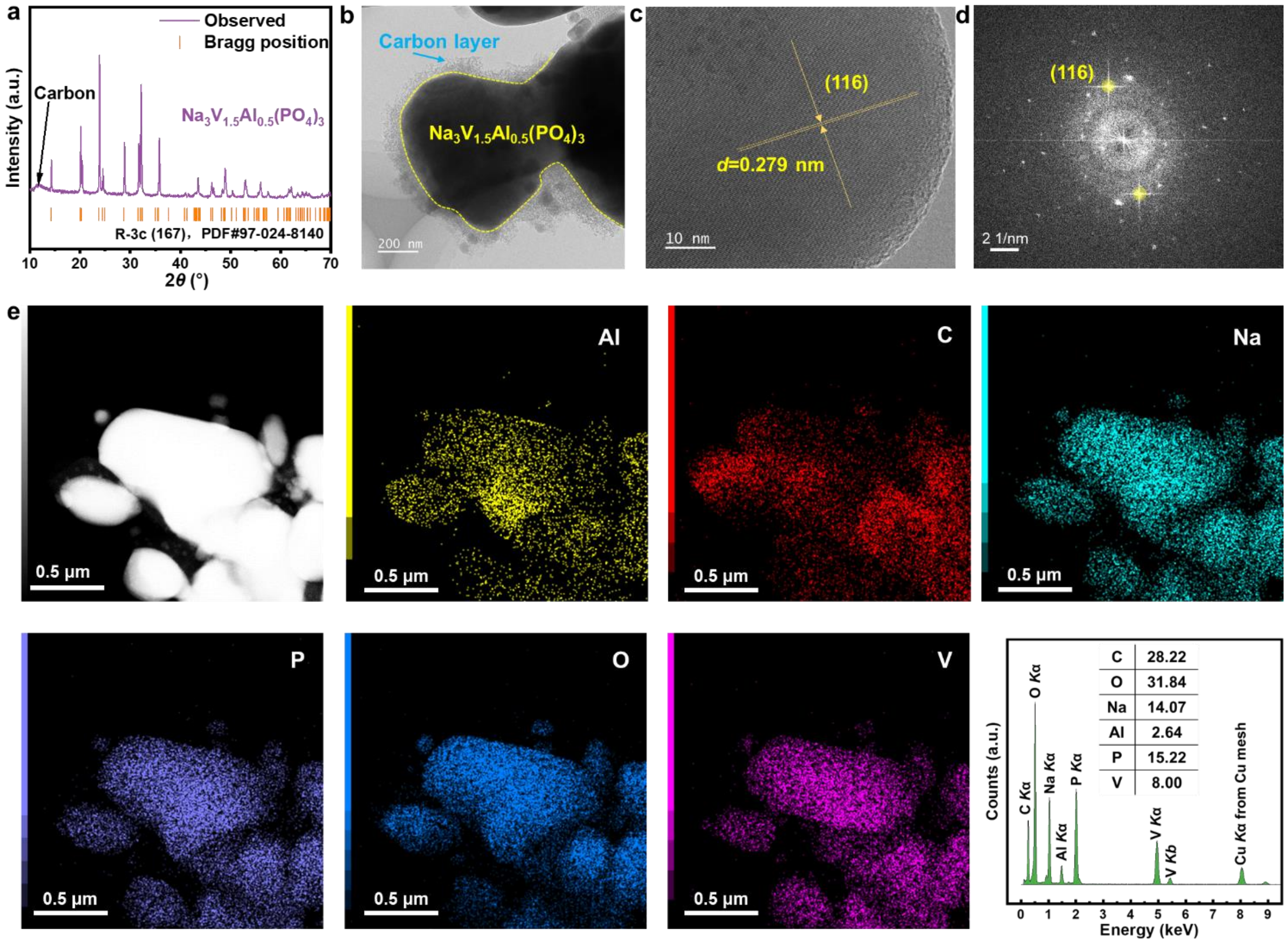

Figure S10. Characterization of the NVAP cathode materials. (a) XRD pattern, (b) low-magnification TEM image, (c) high-resolution TEM image, (d) SAED pattern and (e) HAADF-STEM image with the EDX mapping images and spectrum of Al, C, Na, P, O, V and their contents.

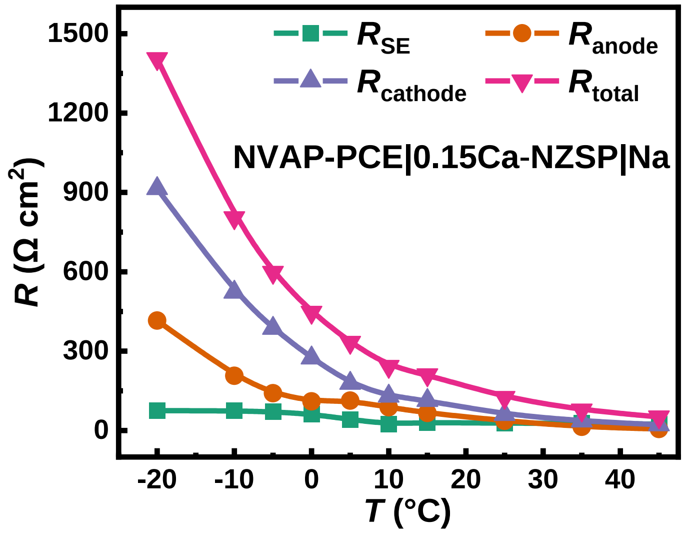

Figure S11. The temperature-dependent resistances of the solid-state NVAP-PCE|0.15Ca-NZSP|Na battery from the solid electrolyte (R_{SE}), the anode interface (R_{anode}) and the cathode interface ($R_{cathode}$) in the temperature range of -20-45 °C.

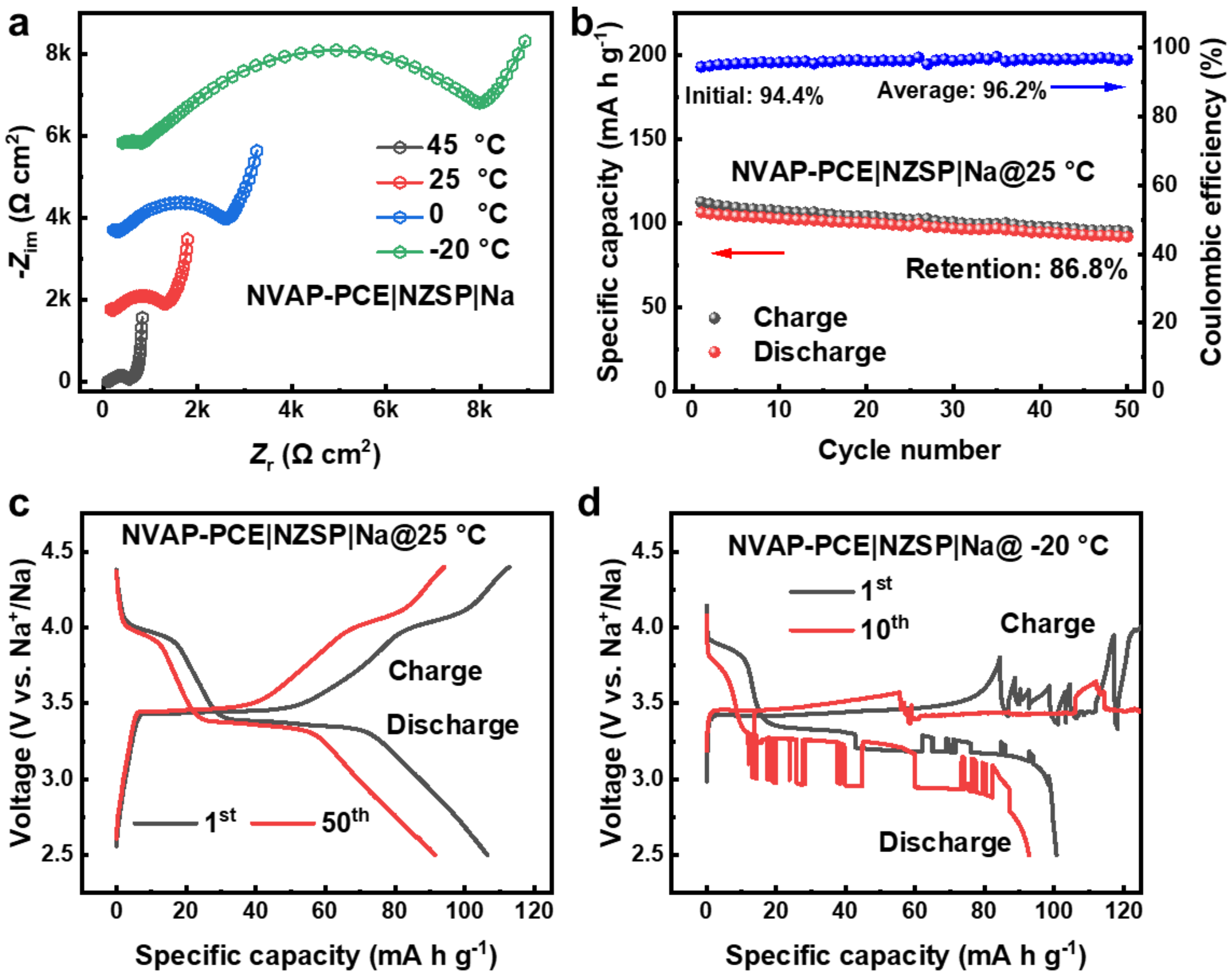

Figure S12. The temperature-dependent resistances of the solid-state NVAP-PCE|0.15Ca-NZSP|Na battery from the solid electrolyte (R_{SE}), the anode interface (R_{anode}) and the cathode interface ($R_{cathode}$) in the temperature range of -20-45 °C.

Table S1. Comparative electrochemical performance of solid-state Na metal batteries using $Na_3Zr_2Si_2PO_{12}$-based solid electrolytes at various test temperatures.

Cell configuration	*T* (°C)	Cycling performance	Rate performance	Ref.
$Na_3V_2(PO_4)_3$-LE|$Na_3Zr_2Si_2PO_{12}$-TiO_2|Na	25	0.1 C/70.6% after 60 cycles	NA	[20]
$Na_3V_2(PO_4)_3$-PCE|SnO_x-$Na_{3.2}Zr_{1.9}Ca_{0.1}Si_2PO_{12}$|Na	25	1 C/98.13% after 450 cycles	4 C/80.5 mA h g^{-1}	[3]
$Na_3V_2(PO_4)_3$-LE|$Na_3Zr_2Si_2PO_{12}$-SPAN|Na	25	0.5 C/87.5% after 200 cycles	2 C/81.1 mA h g^{-1}	[19]
$Na_3V_{1.5}Cr_{0.5}(PO_4)_3$-PCE|TS-$Na_3Zr_2Si_2PO_{12}$|Na	25	100 mA g^{-1}/73% after 400 cycles	100 mA g^{-1}/103 mA h g^{-1}	[13]
$Na_3V_2(PO_4)_3$-LE|$Na_3Zr_2Si_2PO_{12}$|Na-UW	25	0.1 mA cm^{-2}/89.8% after 900 cycles	0.5 mA cm^{-2}/93 mA h g^{-1}	[14]
$NaCrO_2$-PCE|$Na_{3.4}Zr_{1.8}Mg_{0.2}Si_2PO_{12}$|Na	25	1 C/87% after 1750 cycles	5 C/102.6 mA h g^{-1}	[21]
$NaCrO_2$-PCE|$Na_{3.4}Zr_{1.8}Cu_{0.2}Si_2PO_{12}$|Na	25	5 C/86.5% after 660 cycles	10 C/75 mA h g^{-1}	[22]
$Na_3V_{1.5}Cr_{0.5}(PO_4)_3$-PCE|ZBO-$Na_3Zr_2Si_2PO_{12}$|Na	25	10 C/81% after 560 cycles	10 C/108 mA h g^{-1}	[11]
$Na_3V_{1.5}Al_{0.5}(PO_4)_3$-PCE|$Na_3Zr_2Si_2PO_{12}$-CuO|Na	25	5 C/99.0% after 2250 cycles	10 C/111.7 mA h g^{-1}	[23]
$Na_3V_2(PO_4)_3$-LE|$Na_{3.4}Zr_{1.6}Sc_{0.4}Si_2PO_{12}$|Na	80	1 C/98.7% after 50 cycles	NA	[24]
	50	1 C/99.8% after 100 cycles	Na	
	30	1 C/90.9% after 300 cycles	1 C/108.5 mA h g^{-1}	
	0	0.1 C/89.4% after 100 cycles	NA	
$K_2MnFe(CN)_6$-LE|$Na_3Zr_2Si_2PO_{12}$|Na	25	1 C/83% after 400 cycles	3 C/114 mA h g^{-1}	[25]
	0	1 C/85.8% after 280 cycles	1 C/100 mA h g^{-1}	
**$Na_3V_{1.5}Al_{0.5}(PO_4)_3$-PCE|0.15Ca-NZSP|Na**	**45**	**0.5 C/97.1% after 50 cycles**	**0.5 C/122 mA h g^{-1}**	**This work**
	**25**	**0.5 C/97.2% after 50 cycles**	**0.5 C/112 mA h g^{-1}**	
	**0**	**0.5 C/95.0% after 275 cycles**	**0.5 C/102 mA h g^{-1}**	
	**-20**	**0.5 C/99.1% after 50 cycles**	**0.5 C/82 mA h g^{-1}**	

Note: LE=liquid electrolyte, 1 M $NaClO_4$ in v/v 1:1 EC:PC with 5% FEC; PCE=plastic crystal electrolyte; SPAN=sulfurized polyacrylonitrile; TS=two-step sintered; UW=ultrasonic welding; ZBO=zinc borate.

References

[1] C. Sun, Y. Zhao, Q. Ni, Z. Sun, X. Yuan, J. Li, H. Jin, *Energy Storage Mater.* **2022**, *49*, 291-298.

[2] H. Gao, L. Xue, S. Xin, K. Park, J. B. Goodenough, *Angew. Chem. Int. Ed.* **2017**, *56*, 5541-5545.

[3] Y. Lu, J. A. Alonso, Q. Yi, L. Lu, Z. L. Wang, C. W. Sun, *Adv. Energy Mater.* **2019**, *9*, 1901205.

[4] P. Hohenberg, W. Kohn, *Physical Review* **1964**, *136*, B864-B871.

[5] W. Kohn, L. J. Sham, *Physical Review* **1965**, *140*, A1133-A1138.

[6] G. Kresse, J. Furthmüller, *Physical review B* **1996**, *54*, 11169.

[7] P. E. Blöchl, *Physical Review B* **1994**, *50*, 17953-17979.

[8] J. P. Perdew, K. Burke, M. Ernzerhof, *Physical Review Letters* **1996**, *77*, 3865.

[9] S. Grimme, J. Antony, S. Ehrlich, H. Krieg, *J Chem Phys* **2010**, *132*, 154104.

[10] H. J. Monkhorst, J. D. Pack, *Physical Review B* **1976**, *13*, 5188-5192.

[11] C. Wang, C. Sun, Z. Sun, B. Wang, T. Song, Y. Zhao, J. Li, H. Jin, *J. Mater. Chem. A* **2022**, *10*, 5280-5286.

[12] Y. Zhao, C. Wang, Y. Dai, H. Jin, *Nano Energy* **2021**, *88*, 106293.

[13] C. Wang, H. Jin, Y. Zhao, *Small* **2021**, *17*, 2100974.

[14] X. Wang, J. Chen, D. Wang, Z. Mao, *Nat. Comm.* **2021**, *12*, 7109.

[15] Z. Zhang, S. Wenzel, Y. Zhu, J. Sann, L. Shen, J. Yang, X. Yao, Y.-S. Hu, C. Wolverton, H. Li, L. Chen, J. Janek, *ACS Appl. Energy Mater.* **2020**, *3*, 7427-7437.

[16] Z. Gao, J. Yang, H. Yuan, H. Fu, Y. Li, Y. Li, T. Ferber, C. Guhl, H. Sun, W. Jaegermann, R. Hausbrand, Y. Huang, *Chem. Mater.* **2020**, *32*, 3970-3979.

[17] Y. Uchida, G. Hasegawa, K. Shima, M. Inada, N. Enomoto, H. Akamatsu, K. Hayashi, *ACS Appl. Energy Mater.* **2019**, *2*, 2913-2920.

[18] H. Fu, Q. Yin, Y. Huang, H. Sun, Y. Chen, R. Zhang, Q. Yu, L. Gu, J. Duan, W. Luo, *ACS Mater. Lett.* **2019**, *2*, 127-132.

[19] X. Miao, H. Wang, R. Sun, X. Ge, D. Zhao, P. Wang, R. Wang, L. Yin, *Adv. Energy Mater.* **2021**, *11*, 2003469.

[20] J. Yang, Z. Gao, T. Ferber, H. Zhang, C. Guhl, L. Yang, Y. Li, Z. Deng, P. Liu, C. Cheng, R. Che, W. Jaegermann, RenéHausbrand, Y. Huang, *J. Mater. Chem. A* **2020**, *8*, 7828-7835.

[21] C. Wang, Z. Sun, Y. Zhao, B. Wang, C. Shao, C. Sun, Y. Zhao, J. Li, H. Jin, L. Qu, *Small* **2021**, *17*, 2103819.

[22] D. Li, C. Sun, C. Wang, J. Li, Z. Wang, H. Jin, *Energy Storage Mater.* **2023**, *54*, 403-409.

[23] R. Miao, C. Wang, D. Li, C. Sun, J. Li, H. Jin, *Small* **2022**, *18*, 2204487.

[24] Q. Wang, C. Yu, L. Li, X. Liu, X. Zhang, G. Gao, Y. Wang, G. Li, *Energy Storage Materials* **2023**, *54*, 135-145.

[25] Q. Ni, Y. Xiong, Z. Sun, C. Sun, Y. Li, X. Yuan, H. Jin, Y. Zhao, *Adv. Energy Mater.* **2023**, *13*, 2300271.
